# EVALUATION OF A TELEPHONE-BASED PROGRAM DESIGNED TO REDUCE LONELINESS IN OLDER VETERANS

**DOI:** 10.1093/geroni/igz038.1153

**Published:** 2019-11-08

**Authors:** Christine E Gould, Christine Juang, Terri Huh, Sowmya Iyer

**Affiliations:** 1 VA Palo Alto Health Care System, Palo Alto, United States; 2 VA Palo Alto, Palo Alto, California, United States; 3 San Francisco VA Health Care System, San Francisco, California, United States

## Abstract

We examined the benefits and challenges of implementing a program aiming to reduce older Veterans’ loneliness. The program helped connect older Veterans with others through telephone-based group activities. Thirty-four Veterans were enrolled in the program, of which 14 attendees called in to one or more activities. Data were collected at baseline, 3-months, and 6-months. Differences in baseline characteristics between attendees and non-attendees were calculated. Changes in Veterans’ perceived loneliness, depression, anxiety, and quality of life were evaluated. Client satisfaction were analyzed. Qualitative data analyses were conducted to identify perceived benefits and challenges of participating in this program. Attendees were more likely to be depressed and have limited environmental resources to promote quality of life than non-attendees. A significant decrease in loneliness was found among attendees from baseline to 3-months. Overall, Veterans were satisfied with this program and the services provided. Perceived benefits included a structured program with interesting topics to spend time on as well as the opportunity to socialize, exchange ideas, and connect with other Veterans. Perceived individual challenges (e.g., hard of hearing) and program-level challenges (e.g., complicated procedures) were observed. Results demonstrated that this telephone-based program is feasible and beneficial for older Veterans to reduce loneliness. Several barriers and challenges were noted, providing insight into ways to improve the implementation of this program.

